# Loss of ancestral food practices and perception of its effect on children’s health among Inga indigenous grandmothers, Nariño, Colombia

**DOI:** 10.1186/s12889-022-13828-z

**Published:** 2022-07-30

**Authors:** Claudia Amaya-Castellanos, Edna M. Gamboa-Delgado, Etelvina Santacruz-Chasoy, Blanca E. Pelcastre-Villafuerte

**Affiliations:** 1 Departamento de Salud Pública, Escuela de Medicina, Universidad Industrial de Santander, Carrera 32 #29-31, Bucaramanga, Colombia; 2 Medical Anthropology Research Center, Universitat Rovira i Virgili, Tarragona, Catalunya Spain; 3 Escuela de Nutrición, Universidad Industrial de Santander, Bucaramanga, Colombia; 4 Centro de Investigación en Sistemas de Salud, Instituto Nacional de Salud Pública, Cuernavaca, México

**Keywords:** Ancestral food, Indigenous, Children’s health, Intercultural health

## Abstract

**Objective:**

Novel foods and dietary practices, a lack of available land, and displacement by armed conflict have affected the ancestral food traditions practiced by the Inga community in Aponte, in Nariño, Colombia. These factors have led to problems with food security and malnutrition, which have impacted the growth and development of children. Therefore, this study is aimed at identifying the changes in ancestral food practices reported by Inga grandmothers, and the possibility of recuperating them in order to improve children’s health.

**Method:**

A qualitative study was conducted that included 24 mothers with children under five years old and 25 grandmothers in nine Inga communities. Participants were recruited using snowball sampling. Free listing was used to identify changes in food patterns, and semi-structured interviews were conducted with 20 grandmothers to delve deeper into the subject. A translator of the Inga language facilitated communication, and the Inga researcher validated the translation using audio recordings. Each interview was transcribed and categorized for the purpose of analysis, using the NVivo 12 software.

**Results:**

Free lists showed changes from a corn-based to a rice-based diet and a wide variety of non-ancestral food products. According to the grandmothers, “tiendas” have replaced traditional foods with those that are easy to prepare, which are attractive to mothers as well as to the children because of their flavor. Ancestral practices such as grinding, peeling, and log cooking are being abandoned. Government programs and daycare have incorporated new food that compete with traditional ones, with no clear evidence of an intercultural approach. Added to this is the dismissal by young mothers of the knowledge held by their grandmothers, which hinders the continuation of traditions.

**Conclusions:**

The findings suggest that it is necessary to prevent the loss of the Inga food culture, and policies need to be created that promote and protect ancestral knowledge and that help to regain the value of the “chagra” farming system, with the support of elders, authorities who are recognized by the community, and government technicians, as recommended by the grandmothers who participated in this study.

## Introduction

Food practices of many Indigenous peoples are strongly linked with their ways of interpreting and seeing the world. Altering these practices impinges on the way of life of some community members [1]. Various ancestral practices have changed over the years, beginning with the European colonization of the Americas [2]. As time passed, several factors have decreased the consumption of the communities’ ancestral foods, which are foods that belong to a specific region and to the gastronomic heritage of that region, given the value that they contribute in terms of both preparation and consumption. For this reason, ancestral foods require knowledge and generate cultural identity [3, 4]. Determinants of the loss of ancestral foods include the exhaustion of natural resources, the replacement of native crops by commercial ones, and changes in farming practices. These determinants pose a risk to the food security of these communities [5]. In Colombia, roughly 50.2% of Indigenous households have subsistence crops [6].

Reports have shown that problems with food insecurity and the “double burden of malnutrition” (simultaneous presentation of undernutrition and overweight/obesity) are greater for Indigenous populations than for non-Indigenous populations [7, 8]. In Colombia, 77% of Indigenous households are food insecure, compared with 52.3% of households in the general population [6].

Furthermore, one age group that requires more attention during a critical life stage is children under 5 years of age, given that eating practices during the first years of life impact growth and development [9]. It is well known that malnutrition hinders learning processes, weakens the immune system and increases the presence of infections [10]. In Colombia, 29.6% of indigenous children under 5 years of age are stunted (low height for their age), compared to 10% of non-Indigenous children. Malnutrition among Colombian Indigenous children in this age group is 7.2%, compared to 3.0% for non-Indigenous children [6].

Food practices have significantly changed for Indigenous groups in both lower- and higher-income countries [11]. For instance, some Indigenous children in Canada have been found to consume fast food and processed foods at least once per week, and roughly half consume candy, desserts and packaged foods with high salt contents at least once per day [12]. In the case of the Embera Indigenous community in Colombia, their ancestral practices such as hunting, fishing, and gathering have become unsustainable due to low productivity and socioeconomic conditions. This is reflected in deficiencies in folic acid (34.2%), calcium (93%), vitamin A (61.4%), and zinc (75.7%), as well as chronic malnutrition (68.9% of children under 10 years of age) [13]. Meanwhile, other studies in northern Colombia reported that 13.9% of Arhuacos children were overweight and 8.8% were obese [14]. In 2015, roughly 5% of Colombian Indigenous children under 5 years of age were obese [6].

Actions to address these nutritional problems have had poor results in some countries with large Indigenous populations [15]. A good deal of this problem is due to a lack of epidemiological indicators to better evaluate the situation [16]. Added to this concern is a process of cultural disconnection that has occurred in some of the communities, in which practices that are foreign to the Indigenous culture are more highly valued than the knowledge of Elders, resulting in the partial or total replacement of food traditions [1]. Given the reality of these communities, this situation suggests that an attempt should be made to complement ancestral diets with foods from other contexts but with nutritional value, and to consider some modern practices that facilitate their preparation.

One population experiencing problems related to changes in eating practices is the Inga community. This is one of 102 Indigenous populations that still exist in Colombia, and it represents 1.1% of the country’s Indigenous population [17]. In 2018, there were 19,561 people who self-identified as belonging to the Inga community [16], 62.4% of whom lived in the department of Putumayo, followed by Nariño (16.6%) and Cauca (4.4%) [17].

Ever since Ingas arrived in Aponte, Nariño in 1700 AD, they have had difficulties defending their territory. Their territory was colonized in 1930, and since then “mestizaje” process have led to a weakening of cultural values (language, dress, food, thought, and spirituality). This brought about the appearance of money, the “tiendas” (small informal markets offering everyday consumer products), alcohol consumption, and even prostitution [18]. In 2003, the Inga people began a process to strengthen their cultural identity and to eliminate elements that were foreign to their culture, which they believe have deteriorated the lives of the Inga. To address the negative effects of globalization and colonization and to be able to continue existing as an Indigenous people, they implemented the principle of “Living Well.” This is a strategy to preserve cultural uses and customs, strengthen Indigenous authorities, and reduce sources of contamination to protect the water and care for the plants and animals [19].

Since the 1970s, several factors have affected the quality of the soil as well as routine “chagra” practices [18] —a farming system that ensures Indigenous food security and represents their worldview [20]. Farming difficulties have included indiscriminate deforestation for the extraction of wood, and since the 1970s, the presence of guerrilla and paramilitary groups. During the 1980s and 90s, the cultivation and trafficking of poppy was related with aerial spraying with glyphosate [18], ordered by the Colombian government to combat illicit crops. And in 2015, a geological fault caused a crack that divided the territory, destroyed houses, and made it difficult to walk in the municipality [18].

In addition, the arrival of “Western” foods (an expression used by the grandmothers to indicate foods that are not of Inga origin) was related with children’s preferences for foods sold in “tiendas” or received in children’s daycare centers. Most young Inga have abandoned agricultural activities and ancestral foods, looking for prestige when buying and consuming “Western” foods [19]. With the passage of time, adults have happened to prefer “Western” food because of the speed and ease of cooking [19]. All these factors have decreased food security for the Inga people.

Faced with this concern, one of the coinvestigators of this study, a member of the Inga people, expressed her interest in addressing the problems in her community. She joined the other researchers to explore changes in the food and nutrition of young Inga children. Therefore, the study herein was aimed at identifying the replacement of food practices among children under 5 years old based on conversations with grandmothers belonging to the Inga community in Aponte (Nariño, Colombia). It also explored the perception of health effects and the possibility of recovering ancestral food practices, in order to improve the community’s food security and the health of under 5-year-old children. This study is based on the grandmothers’ key role as knowledge-holders of their community and of their culture’s traditions, including their food practices.

## Methods

A phenomenological qualitative study [21] was carried out with mothers and grandmothers of children under 5 years of age, from the nine communities in the Inga Indigenous reservation in Aponte, Nariño, respecting intercultural agreements with the Inga people. The children’s current food practices were explored based on the self-reflection and knowledge of Inga grandmothers and mothers. The participants included 24 mothers from the nine communities in the Inga Indigenous reservation in Aponte, Nariño. The median age of the mothers was 29 years old (Q_25_: 25 years, and Q_75_: 35 years) and the median number of children was 2. Most of the mothers interviewed (46.15%) had incomplete secondary education. The occupation reported most frequently (76.92%) was homemaker.

In addition, 25 grandmothers were invited to participate, most of whom were grandmothers of those children. Their median age was 66 years old (Q_25_: 57 years old, and Q_75_: 75 years old), the median number of children was 4 and the median number of grandchildren was 5. Only 5 of the grandmothers had primary education, and the others had incomplete primary or did not have formal education.

The study sought the participation of at least one grandmother and one mother from each community. The snowball technique was used to identify participants [22], beginning with some key participants on the reservation (mothers identified by the Inga researcher), and then intentionally including other women.

Free lists were used to identify changes in the foods that the mothers and grandmothers fed to their under 5-year-old children. This technique made it possible to obtain systematic data about a cultural domain by using a set of organized words, concepts, and phrases [23]. A cultural domain is a knowledge shared by a social group, built in a tacit manner, which allows its members to interpret common experiences [24]. To this end, the mothers were asked to make a list of the foods that they frequently fed to their under 5-year-old children, and the grandmothers were asked to list the foods that they typically fed to their children when they were raising them. The foods were recorded in the order mentioned. According to this technique, people refer to each element based on their familiarity with it and its relevance in their context. The free lists were administered in 10 min or less. Once the lists were obtained from all the participants, they were entered into a digital note block file and categorized. Anthropac 4.98 [25] was used to process the data and to obtain the relevance of the responses.

After the data was categorized and processed, salience values were obtained for each free list (Table 1). Salience is understood as the level of importance in terms of presence and position, in this case, of one food with respect to the others [26]. That is, the relevance of the foods given during childrearing, the frequency with which they were mentioned, and the position in which they were recorded were identified according to the cultural domain of the grandmothers and mothers, as expressed by the technique itself [26]. In this case, a salience near 1 indicates that the food mentioned is most important, since it was reported by many people and was one of the top foods listed.

**Table 1:** Food that grandmothers fed to their children in their childrearing years, and that mothers fed to their under 5 years-old children. Results from free lists

Grandmothers (*n* = 25)				Mothers (*n* = 24)			
**Item**	**%**	**Average Rank**	**Salience**	**Item**	**%**	**Average Rank**	**Salience**
Corn	92	4.83	0.639	Rice	88.5	4.57	0.528
“Mote” (boiled grain)	60	2.87	0.514	Soups	76.9	3.7	0.52
Calabaza (*Cucurbita pepo*)	68	5.88	0.437	Coffee	42.3	3	0.341
Collard greens	72	6.44	0.403	“Coladas” (a hot thick drink)	46.2	2.92	0.334
Soups	56	7.07	0.304	Bread	34.6	2.67	0.28
“Arracacha” (Arracacia xanthorriza)	56	7.36	0.301	Eggs	50	4.92	0.259
Beans	48	6.58	0.261	Tortilla	34.6	3.89	0.23
Wheat	40	5.9	0.255	“Aguapanela”	34.6	4.89	0.197
Cane sugar	36	6.11	0.222	Beans	42.3	6.73	0.178
“Coladas” (a hot thick drink)	44	7.82	0.214	Peas	30.8	6.38	0.13
Barley	36	7.44	0.19	Meat	34.6	6.89	0.123
“Aguapanela”	32	7	0.171	Bananas	15.4	3.25	0.116
Cow milk	32	8.13	0.167	Corn	15.4	5.25	0.096
Fava beans	28	7	0.157	Cow milk	11.5	2	0.092
Corn “envueltos”	32	7.75	0.152	Chicken	19.2	5.2	0.091
Peas	28	7.57	0.143	Stew	11.5	4	0.084
Flavored water	36	8.67	0.139	Potatoes	26.9	8.43	0.078
Olloco	32	9.5	0.138	Lentils	19.2	7	0.078
Tortilla	20	5	0.138	Oranges	15.4	5	0.077
“Chicha”	24	8.5	0.121	Apples	11.5	4	0.073
“Cuy” (Cavia porcellus)	28	7.86	0.112	“Guineo” *(*Musa paradisiaca*)*	11.5	4.33	0.069
Sweet potato	24	8.67	0.101	Papaya (Carica papaya)	7.7	3	0.051

Total of 22 of 51 foods reported by grandmothers and 53 reported by mothers

To delve more deeply into the topic and provide an interpretation of the results of the free lists, 20 of the grandmothers were interviewed, given their knowledge about their community and its ancestral and cultural food traditions. The semi-structured interviews were conducted with those who filled out the lists and who wanted to be interviewed and easily expressed themselves. The interview guide explored the following four themes: (1) changes in food practices by children under 5 years old, (2) reasons for those changes, (3) perception of good health status of the children and (4) options for recovering ancestral food practices. These themes served as a priori categories of analysis. The phenomenological perspective [21] was used to analyze the verbal contents of the Inga grandmothers with the aim of identifying the essential structures that give meaning to their perceptions, knowledge, and experiences. The interviews lasted from 20 to 45 min and each one was transcribed and categorized for analysis with the aid of the NVivo program [27]. The information analyzed was verified by the researchers.

A psychologist with expertise in free lists and interviewing conducted these two techniques in the women’s homes. An Inga language translator from the community facilitated communication with non-bilingual grandmothers (Spanish and their Indigenous language). All participants gave their approval to participate and to record the interviews, through informed consent. Their anonymity was protected by assigning codes to the free lists and to the audio recordings of the interviews.

 The study’s protocol was approved by the Universidad Industrial de Santander’s Ethics Committee, and it was performed in accordance with the Declaration of Helsinki and the Colombian legislation on research with humans.

## Results

### Free lists

In all, the grandmothers mentioned 51 foods and the mothers mentioned 53. Table 1 shows the top 22 foods mentioned, although the analysis was performed with the total foods from both groups. The key results show that corn was the food that the grandmothers fed most to their children when they were under 5 years old (90%), and it was placed near the top of the list, roughly in fifth place. The next was “mote” (boiled grain, a type of corn different from the traditional corn), which was reported by fewer people than those who mentioned corn (60%) but was in third place, on average. The food mentioned by the grandmothers that had the third highest salience was zucchini. For the mothers, rice was the food that they fed most to their children, with nearly 89%, and it was in fourth place, on average. That was followed by soups and coffee, which were in third place, on average, though more people mentioned soup (roughly 77%) than coffee (roughly 42%). In addition to the above results, important differences in food groups stand out, which are summarized in Table 2.

**Table 2:** Food groups fed by grandmothers and mothers to under 5 years-old children

Results	Grandmothers	Mothers
Fruits	None reported	Various reported, but not near the top of the lists. Bananas and oranges were reported most (15%).
Vegetables	Several at the top of the list with high salience: zucchini, collard greens, and “arracacha” (Arracacia xanthorriza). Between 56 and 68% reported.	Few reported, only 30% mentioned peas but with low salience.
Cereals	In addition to corn (92%), wheat (40%) and barley (36%) were reported. The latter two with low salience.	In addition to rice (88.5%), bread was reported (36%). Corn was not near the top of the list, with 15% reporting and low salience. Barley was among the last on the list. Wheat not reported. Oatmeal reported but by less than 8%.
Meat	Little reporting of meat (4%), only “cuy” (28%) but with low salience, likewise for hens. Sixth to seventh position.	Meat was reported (34%). Chicken, hens, and sardines reported by less than 19%, with low salience. Fifth to sixth position.
Legumes	Only beans (48%) and similar foods such as fava beans and **“**chirilla” beans. Higher salience in sixth position.	Beans (42%) and lentils (19%) with low salience and between sixth and seventh positions.
Drinks	Cow milk and “aguapanela” equally reported (32%) though the latter had a higher salience. “Chicha” and cow milk given the same position, though the latter had a lower salience and was reported less (24%).	Cow milk mentioned by only 11% with a lower salience than coffee, which was reported by 42%, followed by “aguapanela” (34.6%). Both with a higher salience and in higher positions than cow milk.
Eggs	Eggs reported by 16% and had a lower salience than cow milk.	Eggs reported by 50% and had a higher salience than cow milk.

Summary based on the total foods reported: 51 reported by grandmothers and 53 by mothers

### Interviews

The findings from the interviews that were conducted with the 20 grandmothers in the community are presented below, for each of the four categories analyzed. Some of the testimonies from the Indigenous grandmothers are included in order to better understand each category. Tables 3 and 4 summarize the details of the categories and subcategories.

**Table 3:** Category: Changes in food practices for children under 5 years-old

Type of change	Explanation	Testimony
Abandonment of corn and its derivatives	- Mothers and children prefer rice, bread, and wheat flour for making tortillas and pastas; they abandoned corn-based foods as *tostados* (oven-toasted corn tortilla), “envueltos” (a traditional dish of masa wrapped in corn-husks), and “coladas” (a hot thick drink).	“… we used to give our children corn soup with collard greens and beans. Arracacha, zucchini, C, mote, beans, and mote with lots of beans. That’s what we used to eat before. That’s what we used to give the children. Now all they want to eat is things from the store, noodles, rice, potato chips…” (Grandmother 59 years-4 sons-5 grandsons)
Current predominance of rice and bread in food	Mothers and children prefer to mix rice with eggs, chicken, sardines, or potato chips. They prefer bread, which has replaced the consumption of corn “envueltos”, beans, collard greens, “arracacha” (Arracacia xanthorriza), zucchini, and peas	
Perception of less natural and healthy feeding than before	Increase in the use of salt and Knorr® bouillon cubes Different preparations with “Manteca” and oil. In the past tortillas were not fried but rather toasted over flame	“…before, everything used to be prepared without salt. When you would go, they would have a bundle of salt like this next to the stove, it was that type of coarse salt. When you got there, they would put a grain of salt in the spoon and mix it that way. But it was food without anything, no salt.” (Grandmother 73 years-8 sons-4 grandsons)

**Table 4:** Category: Perception of children’s good health status

Type of change	Explanation	Testimony
Currently the children get sick more than before	Currently, children and mothers in pregnancy consume “scrap” food	“Before, food was very natural. When someone was pregnant, people would eat meat, an animal that had been raised here. Now they eat a lot of “junk” food, sausage, hot dogs, those canned foods. That chicken that comes stored, I don’t know for how many days.” (Grandmother 60 years-6 sons-5 grandsons)
Currently foods have fertilizers or chemicals	Those who consume them get sick, weaken, do not resist diseases, grow rapid but not strong. When they are adults they will have cancer	“In some parts they use a lot of chemicals now. They fumigate so the produce fills out, so it grows. All of that with chemicals. They fumigate the corn now too. The yuca, I see that they learned to use fertilizer with chemicals, and because of that everything is different. The children grow fast, but because of that they are no longer getting old so fast. Why? Because we are eating with chemicals. Everything is with chemicals. Even we are eating chemicals.” (Grandmother 59 years-1 sons-2 grandsons)

#### Changes in food practices for children under 5 years old

In this category, three important changes were identified (see Table 3). The first relates to the consumption of corn. Fourteen of the 20 Inga grandmothers mentioned that when they were moms, theirs and their children’s diets were corn-based, and the food was almost exclusively made with corn. However, 5/20 reported that they were poor and that some days they ate little or did not eat. Currently, mothers have replaced corn with other foods that are more attractive to them and their children, such as rice, bread, flour to make arepas, and pastas. The researchers consider this attraction to stem from the novelty of those foods, from their having been non-existent in the past.

A second change was the inclusion of rice and bread as staple foods. For 18/20 grandmothers, rice is a food that children currently enjoy alone or accompanied with other foods. They noted that feeding children rice is a typical practice by today’s mothers that has resulted in rice replacing traditionally consumed foods. For example, traditional drinks such as “chicha*”* (the original recipe for a fermented corn drink) has been replaced by coffee, which they like to drink with bread.

The third change is the perception that the food eaten is less natural and healthy. For instance, 11/20 grandmothers mentioned that mothers currently use oil and lard in their preparations, and 8/20 mentioned the use of condiments to give flavor to meals. There has been a notably rapid increase in the use of salt and Knorr® bouillon cubes (a brand containing monosodium glutamate used to flavor certain soups). Grandmothers never used these condiments to prepare food.

#### Reasons for changes in food practices

According to the grandmothers, traditional foods were abandoned as a consequence of three factors (see Table 5):

**Table 5:** Category: Reasons for changes in food practices according to the Inga grandmothers

Type of change	Explanation	Testimony
Availability of time and practicality of cooking for the mothers	To make preparing foods easier: - Gas is used instead of wood - No peeling, no grinding - Foods that are quick and easy to prepare.	“The youth of today is lazy. They replace corn with pasta (laughs), they’d rather cook noodles and not grind corn or wheat”. (Grandmother 66 years-2 sons-3 grandsons) “They don’t want to peel mote (boiled grain) or cook because they see that everything is about going to buy at the market and that’s it. They buy Promasa (brand of corn dough that is ground and packaged) for the daughter-in-law. Grind? Her? She says that’s hard…” (Grandmother 65 years-6 sons-6 grandsons)
Children’s food preferences	Foods and attractive preparations: - “Tiendas”, particularly “junk” food, as fried potatoes, candies, or cookies. - Daycare, schools and/or government programs offer rice, juice, meat, eggs, and a variety of preparations	“Nowadays, for them the food at daycare is better, well, they say they have meat there, they have eggs, juice, fruits, everything. But at home there isn’t any of that. They don’t want to eat the foods that used to be eaten anymore.” (Grandmother 74 years-12 sons-10 grandsons)
Changes in the environment	Consumption and food preparation practices have changed because: - the presence of “tiendas” - the availability of money from poppy crops and the extraction of wood - loss of interest in planting crops - preference for better-paying crops - factors that have affected the soil	**“…**before there were those illegal crops, there was a lot of food, all kinds, even the elderly ate well (laughs). So the kids learned to eat that way. Now, these days, they no longer want to eat the food that there is, our food, “fierita” food as they say…” (Grandmother 59 years-4 sons-5 grandsons) “We had a nice plot for planting…We knew how to plant beets, carrots, cilantro…Everything was planted, cabbage, collard greens, and now, they went down there to kill off the coffee, they felled where we used to plant. We’re on the border here, it’s not possible to plant crops” (Grandmother 66 years-2 sons-3 grandsons)

- **Availability of time and practicality of cooking for mothers.** Fifteen of the 20 grandmothers mentioned that cooking used to be more burdensome. Now they choose things that are easier because, according to the grandmothers, the mothers say they have a lot more housework than before, and some even have activities outside home (i.e. as domestic workers). For this reason, they prefer to cook with gas and not with firewood, which involves a good deal of walking in the mountains to gather the wood. However, high gas costs have led to abandoning cooking traditional foods such as “mote” (boiled grain), since that requires large amounts of fuel. To make their work in the kitchen easier, the grandmothers reported that today’s mothers avoid activities such as grinding, peeling, and preparing foods that require a lot of cooking time, and therefore, they choose to buy products that are easy and quick to prepare, such as pasta, rice, and canned tuna.
- **The children’s food preferences**. According to 12/20 grandmothers, children prefer “junk” food, which is described as food that is not very natural, that comes in packages and cans, and is stored for long periods of time, and which is attractive and competes with the foods that they are given at home. Tastes have also changed due to the presence of certain government programs that are run by entities such as the Colombian Family Welfare Institute (ICBF in Spanish). These programs have introduced foods and a variety of preparations that the children like. It was not possible to obtain these foods in the past because these types of programs did not exist.
- **Changes in the environment**. According to the grandmothers, over time some homes in the community have had more money available to them, and this has led to the abandonment of traditional foods. Seventeen of the 20 grandmothers mentioned the example of poppy crops having generated more money for families who grow this crop, which has allowed them to buy different foods from those that were traditionally consumed.

The same situation occurred with the arrival of the wood industry. Most people began to work in this industry instead of planting crops. Since they received much more money from wood than from their typical crops, they could now buy products in the “tiendas,” or from granaries where a variety of foods arrived because of the money circulating in the region. The “tiendas” became an attractive place for children and adults because of the novelty of the products that they offered, products different from those that were traditionally consumed. It should be noted that vegetables and fruits are not purchased in “tiendas” but are obtained in the traditional street market that is open on Sundays, which is based on “Cambalachi Ingano,“ or a barter system (“trueque”).

The planting of poppy and the exploitation of wood were identified as events that changed food practices and determined a good deal of the crops planted. Eight of the 20 grandmothers noted that coffee is currently planted, and while they see that as positive, the cultivation of other types of foods has been lost. Coffee is a preferred crop because selling it earns more money than selling foods such as barley and wheat, which do not generate much money or for which there is no demand. The few “chagras” that have survived grow pumpkin, “arracacha,” carrots, cabbage, wheat, and barley. Corn crops have also decreased because corn is no longer as important in the diet. When the grandmothers were young, in addition to these foods, beans, potatoes, cassava, peas, cabbage, cane, and medicinal plants were planted.

In addition to the changes mentioned above, geological factors in the region have altered food practices. The last landslide and fissure occurred in 2015 and destroyed the plots and crops that existed at that time. The “tiendas” were not nearby, and there generally was not enough money to buy the products that they sold. Therefore, food was centered only on what could be planted and the consumption of a few animals.

#### Perception of children’s good health status

Two factors mentioned by the grandmothers stand out in this category (Table 4): one related to the health conditions of current children compared to the children of their time and the other related to the factors that generate the current health conditions. Thirteen of the 20 grandmothers noted that while they used to live in poverty conditions, the children did not get sick as often as they do now. They were healthier because they ate more natural foods. Now they tend to eat “junk” food. They said that not only do the children eat this type of food, but the mothers also eat it during pregnancy.

In addition, they mentioned that children used to eat better in the past because the food did not contain fertilizer or chemicals. They noted that their illnesses may be from the presence of those substances in the food. Eleven of 13 grandmothers believed that the chemicals not only make children sick but also make them weaker, and that is why they can’t even “handle” the flu. They also believed that when they grow up they will get cancer.

In the past, the children got sick for very different reasons, such as a stomachache, fever, “looseness” (an intestinal problem characterized by stomach pain and frequent soft and liquid stools), and “worms” (intestinal parasites). People died because of the lack of timely medical care on the part of health care workers or due to a lack of knowledge about how to treat certain types of illnesses. Now they get sick with stomach aches, coughs, the flu and the cold. They mentioned that some children are even born with problems with their bones.

Lastly, they noted (16/20) that children now are not as healthy as before because they are not always happy and active. They complain and cry about pain for no reason, they do not enjoy the food they are given, and some foods hurt them. It should be noted that for 14/20 grandmothers, it was not clear whether the foods that they have access to now are nutritionally better than what they used to feed their children.

#### Alternatives for recovering ancestral food practices

Some (15/10) of the grandmothers’ recommendations for returning to the food practices of their times were to avoid buying foods from the “tienda” and return to cultivating crops (i.e. corn, peas, beans, collard greens, potatoes, cabbage, and fava beans). They also recommended consuming what is planted since that is healthier because it does not contain chemicals, and returning to raising animals for consumption. This includes seeking the help of the community’s authorities to return to raising sheep, since they provide good food and one animal can feed a family for a month. In addition, 13/10 reported that it is very important to educate the children, such as teaching the girls when young the importance of the foods that they used to eat, especially corn and all the ways to prepare it, so that they are accustomed to it when they become mothers. By teaching the children to eat the foods that were eaten in the past they will recognize the taste of traditional cooking from the time they are young.

“It’s the parents who teach them to eat from the time they are little, what is done, what is eaten. Because if you give them nothing but rice or nothing but chicken like that from the ”tienda,” well they will get used to it and they won’t eat anything else anymore. Now if you go to offer them corn soup, they won’t accept it because they weren’t taught that when they were young.” (Grandmother 64 years-2 sons-0 grandsons)

Nearly all (18/20) also believed it is very important to feed the children mother’s milk for a longer amount of time, for over 6 months:

“Well, recommend that, I don’t know...to some family. I have a baby grandson who is seven, eight months old, and he barely breast fed up to six months, and from then on he didn’t want any more, only bottle, that’s all, with that milk that they buy.” (Grandmother 66 years-2 sons-3 grandsons)

Lastly, they proposed returning to customs such as using clay pots and log cooking to improve the flavor of the food, and trying to get the daycares to serve food from the region, including the soups that they used to eat in the past. Although some of the grandmothers said that an “experiment” was done once at the daycares that did not work because the children did not like the flavor of some of the foods, and so they did not continue to prepare them.

Nearly all (19/20) the grandmothers also stated that a key obstacle to carrying out these actions is the young mothers themselves, whose lack of interest is a barrier to recovering the food practices of the old days. They said that the grandmothers’ knowledge is not believed anymore, it is undervalued, and the only belief is in what’s “mote.” The young mothers in the community do not accept advice anymore, nor do they seek the help of older women. Knowledge is not transmitted between one another. The grandmothers believed that this limits their ability to teach what they know, such as helping new mothers continue traditional food practices.

“Recommendations now for the moms, well, I don’t say anything to anyone anymore, because they don’t believe it’s anything. Everything is Western now. But before if someone new something, among the mothers themselves, someone would say: What do you do with your child? What do you give him to eat? They don’t have these conversations anymore. They go to the health center, and they tell them to do this and that, and no matter what you tell them they aren’t going to do it anymore.” (Grandmother 60 years-6 sons-1 grandson)

## Discussion

The grandmothers in this study expressed the need to return to planting crops and raising animals, which not only relates to food security but also to food sovereignty, as do garden projects that are being implemented in other Indigenous contexts [28]. In this way, the value of Indigenous food and environmental systems is recognized as a way to reduce hunger problems, particularly among the children in these communities [29]. The discussion of ancestral food practices refers to foods from locally available natural resources that are culturally accepted, as well as practices and the meanings that they entail [30]. This is based on the principle of food practices that provide nutrients, which can better contribute to healthy conditions than industrial foods.

In terms of nutrition, the degree to which ancestral food practices are suitable is particularly important. The grandmothers’ social representations of consuming the natural foods that they had in the past reflect the importance of valuing the practices that are no longer present in the current generation, which they miss and wish to regain. In Colombia, ancestral products such as corn, potato, yuca, and barley are traditional foods that have been associated with less overweight among children and a lower risk of breast cancer [31, 32]. Moreover, this study measured the body mass index of the children and mothers. The results indicate that 34.62% of the mothers were overweight and 11.54% were obese, whereas 7.69% of the children were overweight or obese. These findings show that mothers currently engage in less physical activity.

Moreover, this study illustrates how certain ancestral food practices by the Ingas have been gradually declining, which reflects what is occurring in most Indigenous communities in the Americas [33]. This is characterized by the progressive substitution and termination of cultural practices, which have led to what some authors call ethnic and cultural genocide [2]. The way in which communities relate to food and changes in the forms of production have explained these changes in food practices [34]. The Inga grandmothers perceived the foods that were eaten in their day as healthier than what the mothers currently feed their under 5-year-old children.

In the case of the Inga people of Aponte, the recovery of ancestral food has many benefits. There is evidence that diets based on corn, beans, and pumpkin allowed the subsistence of Mesoamerican peoples [35], which indicates that macronutrients (proteins and carbohydrates) and micronutrients (calcium, vitamin A and niacin) are sufficient to have an adequate diet [36]. Undoubtedly, it is possible to improve this ancestral diet with foods rich in micronutrients (fruits and vegetables). This would be an additional benefit that would clearly improve health conditions. In regard to the ways of preparing food, since mothers prefer to devote less time and physical effort to culinary practices, any intervention to recover ancestral foods should include modern forms of cooking. The priority is to maintain the nutritional benefits and food security associated with ancestral foods.

Consuming the region’s traditional vegetables and cereals is less common now, according to the grandmothers. Nevertheless, the results of this study show that factors existed such as a lack of fruit, meat, and eggs in the days when the grandmothers were raising their children. These results differ from some studies that report the value of ancestral eating practices in Indigenous communities where there is a high consumption of fruits, vegetables, and animal protein [37, 38]. In this study, the particular conditions of the Inga community involve different social, economic, and political problems that have existed since the 1970s [18], which can explain this inconsistency.

The findings herein are consistent with Indigenous populations around the world incorporating in their diet high-energy foods that are high in salt and fat and low in fiber [12, 39]. Access to these types of food in the Inga region is related with the introduction of rubber in the 1970s and poppy in the 1980s [18]. The money that circulates from those activities has made it possible to acquire other food products from the “tiendas,” which in the past were not spaces belonging to this culture. The foods that are sold, and which are included in what some authors call “food technology,” or industrialized high energy density foods [33], are attractive to the Indigenous population, and especially to children since they are novel and their flavors are different from local foods. This type of food is also attractive to mothers because it is easy to prepare and thereby reduces housework, while it also contributes to the replacement of traditional foods.

The findings suggest that these populations imitate “Western” patterns that they find attractive, which even gives them a sense of belonging in a context where Indigenous populations have historically lacked recognition and have experienced marginalization [40]. Since some Indigenous peoples seek to look like workers or travelers who arrive from other regions, they eat what they see them consume. The results also explain the youth’s lack of interest in the land and their devaluation of the “chagra” farming system, and changes in the mothers’ activities. While mothers continue to raise children and care for their husbands, including engaging in activities related to food preparation, they now express disinterest in receiving advice and teachings from knowledgeable women in their community, and in continuing practices such as peeling, grinding, and log cooking. Since this provides more free time, they can perform other activities at home, or rest, or talk with others.

Older women in other Indigenous contexts in Latin America refer to the lack of interest in their knowledge and advice as devaluation, and thus, a lack of recognition of their ancestral knowledge [41], even though women have historically been linked with both the preparation of food as well as the implementation of production and consumption strategies [42]. In this sense, cultural knowledge about food needs to be transmitted from generation to generation, since this would make it possible to learn about and reinforce the community’s own knowledge and practices, and for that to be reflected in the foods that are fed to the children [43].

The Inga’s consumption of foods that are not part of the Indigenous food tradition is consistent with findings by studies of non-Indigenous populations that have similar socioeconomic characteristics [43]. Studies have found that for groups that have poor food environments, healthy options are not available and obtaining nutrient-dense foods is expensive [44]. While the risk of malnutrition from deficits or excesses may be the same for the general population, the risk is higher in Indigenous communities due to poverty-related food insecurity, the availability of low-cost low-nutrition foods and a lack of resources for production [45].

As has been documented in other Indigenous populations, the Inga grandmothers’ concept of “healthy foods” is associated with what is natural, what comes from the earth, while “non-healthy foods” are products that are produced by industrialization processes [46]. Nevertheless, this is not always the case in Indigenous communities, since some have indicated that consuming industrialized foods is good because it reflects the financial ability to obtain them [33], which reinforces the tendency to imitate “Western” patterns. In addition, according to the grandmothers, healthy foods are associated with what is natural and the presence of chemical substances in foods reduces their healthiness. They reported that ancestral crops have sometimes been fumigated with the glyphosate used to control illegal poppy crops, which has affected food practices in three ways: (1) they do not consume their crops for fear of contamination, (2) they consume what they plant in small quantities because they believe it is not good for their health, and (3) they plant fewer crops because they believe that they affect the quality of the soil. These factors have been described in the literature [47, 48] and are consistent with the perception of the participants in the present study.

Given the situation described above, the community plants fewer crops and favors crops that generate more income. With the money they earn, they can pay to travel to other places, buy medicines, and make household improvements. Ancestral foods are replaced as a result, such as corn —a basic food for Indigenous cultures in most of the Americas, where some countries have seen notable decreases in consumption [39]. For the Ingas, bread, rice, and pasta are widely present and compete with this age-old food. Thus, changes not only involve the types of food consumed and food practices, but also how the Indigenous communities have gone from being producers-consumers to being only consumers, which is characteristic of the West [33]. This can be explained by globalization, which intrudes into these spaces and brings about both the marginalization of small-scale production as well as “indigenization” —a movement to revitalize Indigenous food practices in order to regain control over food and production practices that accord with their own culture [2].

Together, all these findings suggest that the recovery of ancestral practices should consider what was healthy in the past without neglecting current healthy practices or the inevitable permeability of these communities to consuming “Western” products, given the close relationship that currently exists between the cultures. Furthermore, it should be recognized that the value of traditional food practices goes beyond simply consuming food, and that foods can be healthy or of little benefit. Ancestral means seeing the underlying symbolic content and how knowledge gives a community identity [49].

Under this premise, strategies by government social programs need to be strengthened so that they actually take approaches that are both intercultural and intersectoral, and take into account social determinants such as culture and those that generate lags in these communities. This means striking a balance between “good nutrition” from a “Western” perspective and healthy eating based on ancestral knowledge.

Recovering knowledge and documenting ancestral practices by Indigenous communities could contribute to future food security and sustainability for these communities [50], as well as to the subsistence of the population in general, at a time when food production is decreasing in some regions of the world, which poses a risk to the survival of our species [42, 51]. The expected effect is to achieve autonomy to decide what food to produce, consume, and market. All actions that promote cultural strengths and the autonomy of communities will be positive [52], as will those that recognize ancestral practices, including the expression of the knowledge that is held by the communities. These ancestral practices are worth knowing and should be protected, particularly since other cultures have lost contact with natural food sources, especially those with high incomes [30]. In addition, public policies should promote and protect the health of these communities and strengthen ancestral knowledge so that there is a return to planting and traditional production practices, and to valuing the “chagra” farming system. This can be accomplished by relying primarily on the knowledgeable grandmothers, government technicians, and respected people in the community such as elders and community authorities [18], as recommended by the community itself.

With regard to properly interpreting the findings, it is important to mention that the data included in this study corresponds only to what was reported by the grandmothers, as knowledge-bearers. It is known that among Indigenous people, the elders are the holders and transmitters of the knowledge of their ancestors. Specifically, the knowledge and experience of the grandmothers is linked to the development and growth of the children and the reproductive health of women [53]. Since this is consistent with the approach of this study, which sought to recover ancestral food practices, reports from current mothers were not included. The inclusion of their knowledge and opinions after some years have passed will undoubtedly be key to better understanding ancestral practices and the process of acculturation.

## Conclusions

The main finding of this study is that there is a tendency to forget some of the ancestral food practices of the Inga Indigenous people in Aponte. This includes exclusive breastfeeding and the consumption of corn and vegetables such as zucchini, collard greens, and “arracacha” (Arracacia xanthorriza), which have been replaced by the consumption of foods such as rice, pasta, and highly processed low-nutrition industrialized foods, along with increased salt consumption. Some of the reasons for the replacement of these practices are: the amount of time available to mothers for cooking, new food preferences on the part of children, and changes in the environment. The presence of poppy crops and the exploitation of wood have increased the circulation of money, thereby increasing buying power for high-energy low-nutrition foods. In addition, aerial spraying with glyphosate to eradicate poppy crops and landslides have had a geological impact, bringing an end to the cultivation of various crops on the community’s land.

Changes in the food practices of Inga mothers are determined by their history and context. Social, economic, and political factors have led to the modification of cultural patterns, such as diets, among other Latin American Indigenous peoples [54]. The grandmothers perceived the new food practices that have been adopted by younger generations as having had several consequences, including children becoming sick more often, their growth not being optimal, and even the appearance of illnesses that did not previously exist in the community, such as cancer, which may be caused by chemical substances in the foods that are eaten today. To regain food security and sovereignty as well as improve health and wellbeing, the grandmothers recommended returning to planting and to consuming foods such as corn, peas, beans, collard greens, and fava beans, as well as returning to raising animals for consumption, such as sheep. They also recommended teaching the young girls how to prepare traditional foods, returning to exclusive breastfeeding, and recovering the food culture belonging to their own traditional food customs, such as log cooking and using clay pots. While the findings herein cannot be generalized, they are valuable for broadening the sphere of knowledge about the subject, and they serve as a useful tool for supporting both communities and decision-makers who are addressing this problem in communities with similar characteristics.

In conclusion, although there are many experiences that describe ancestral knowledge and its potential benefits, including nutritional value and food security [55, 56], the authors have not found any successful experiences published in the specialized literature. More studies are needed to explore possible interventions to recover ancestral food practices, as they are part of the Indigenous worldview. Furthermore, interventions to improve the nutrition conditions of Inga communities should consider these results in order to improve food security and follow a diet that is more appropriate in terms of nutritional requirements. Undoubtedly, an intervention that considers intercultural aspects will be more effective in the long term than an intervention that incorporates elements that are foreign to the Inga culture.

## Data Availability

The datasets generated and/or analyzed during the current study are not publicly available to protect the privacy of participants but are available from the corresponding author on reasonable request.
